# Safety and effectiveness of surgical fixation versus non-surgical methods for the treatment of flail chest in adult populations: a systematic review and meta-analysis

**DOI:** 10.1007/s00068-021-01606-2

**Published:** 2021-02-06

**Authors:** Ayobobola A. Apampa, Ayesha Ali, Bryar Kadir, Zubair Ahmed

**Affiliations:** 1 Institute of Inflammation and Ageing, College of Medical and Dental Sciences, University of Birmingham, Birmingham, UK; 2 Cancer Research UK Clinical Trials Unit, Institute of Cancer and Genomic Sciences, University of Birmingham, Birmingham, B15 2TT UK; 3 Surgical Reconstruction and Microbiology Research Centre, National Institute for Health Research, Queen Elizabeth Hospital, Birmingham, B15 2TH UK

**Keywords:** Flail chest, Surgery, Surgical procedure, Conservative management, Conservative treatment

## Abstract

**Purpose:**

The objective of this systematic review is to compare the safety and efficacy of surgical fixation of rib fractures against non-surgical interventions for the treatment of flail chest in the adult population.

**Methods:**

A search was performed on the 22nd of July 2020 to identify articles comparing surgical fixation versus clinical management for flail chest in adults, with a description of the outcome parameters (resource utility, mortality, adverse effects of the intervention and adverse progression in pulmonary status). Relevant randomised controlled trials were selected, their risk of bias assessed, and the data then extracted and analysed.

**Results:**

157 patients were included from four studies in the analyses, with 79 and 78 patients in the surgical and non-surgical groups, respectively. The pooled effects of all outcomes tended towards favouring surgical intervention. Surgical intervention was associated with lower rates of pneumonia (*I*^*2*^ = 46%, *Tau*^*2*^ = 0.16, *p* = 0.16), significantly lower rates of tracheostomy (*I*^*2*^ = 76%, *Tau*^*2*^ = 0.67, *p* = 0.02), and a significantly lower duration of mechanical ventilation (*I*^*2*^ = 88%, *Tau*^*2*^ = 33.7, *p* < 0.01) in comparison to the non-surgical management methods.

**Conclusion:**

Our results suggest that surgical intervention reduces the need for tracheostomy, reduces the time spent in the intensive care unit following a traumatic flail chest injury and could reduce the risk of acquiring pneumonia after such an event. There is a need for further well-designed studies with sufficient sample sizes to confirm the results of this study and also detect other possible effects of surgical intervention in the treatment of traumatic flail chest in adults.

**Supplementary Information:**

The online version contains supplementary material available at 10.1007/s00068-021-01606-2.

## Introduction

In those with multiple traumatic injuries, trauma to the thoracic area is very common. Rib fractures are the most common of these traumatic injuries, found in 20% of patients who have suffered thoracic trauma [1]. Flail chest (FC) or flail thoracic segment, is defined as a segment of the chest wall that moves paradoxically relative to the rest of the chest wall during respiration. This life-threatening condition is seen when there is a loss of bone continuity of the flail segment from the rigid thoracic wall around it, due to comminuted costal fractures (at least two adjacent bifocal rib fractures) or dislocations as a result of trauma [2]. Cases of multiple rib fractures, leading to unstable chest wall segments represent high energy traumatic impact with significant rates of morbidity and mortality [3]. This may be attributed to a number of associated complications, such as haemorrhage, pneumonia, acute respiratory distress syndrome and other associated intra- (pulmonary contusion, pneumothorax and haemothorax) and extra-thoracic (skeletal fractures, abdominal injuries and brain injury) injuries [4]. Mediastinal and diaphragmatic injuries are also common.

Surgical stabilisation is appropriate for patients who cannot be weaned off from a ventilator or suffer from persistent pain, chest wall instability and a progressive decline in pulmonary function [5–9]. The aim of surgical stabilisation is to restore thoracic wall stability and normal ventilation [5–9]. It requires the use of metal wires, titanium plaques and bars to stabilise the fractured structures. The fixation can be done intramedullary or external to the fractured bones [10]. Restoration of thoracic wall rigidity and normal ventilation allows for a decrease in rates of morbidity and mortality that may otherwise be due to sepsis, pneumonia and acute respiratory distress syndrome. At the time of this review, no other systematic reviews exist that focus exclusively on the management of FC in adults. This systematic review aimed to gather and analyse all of the current data, exclusively from prospective randomised control trials (RCTs), comparing the surgical approach to treating FC to a conservative, non-surgical approach to management in adults. The results of this review should allow for greater perspective concerning patients’ treatment options after traumatic FC injury and can aid surgeons in their decision to choose between surgical versus non-surgical management techniques.

The primary outcome of this systematic review is the length of stay in the intensive care unit (ICU). The overall length of stay in hospital was also evaluated. The secondary outcomes of this review are the in-hospital mortality, duration of mechanical ventilation and complications such as the adverse effects of the intervention (bone infection, wound infection and pain), and the adverse progression in pulmonary status (tracheostomy and pneumonia).

## Methods

### Literature search

We used the search strategies recommended for the Preferred Reporting Items for Systematic Reviews and Meta-Analyses (PRISMA) reporting guidelines. The following databases were searched on 22nd of July 2020: Cochrane Central Register of Controlled Trials (CENTRAL), PubMed, Scopus, Embase (OvidSP), Medline (OvidSP) and ClinicalTrials.gov. The searched items consisted of terms related to flail chest, surgical fixation and clinical management (for the full search strategy, see Supplementary Table 1). The titles and abstracts were screened and verified by two reviewers, A.A.A and Z.A. Any inconsistencies were resolved following discussions between authors.

### Inclusion and exclusion criteria

Studies were only included if they meet the following inclusion criteria: (1) prospective  RCTs, (2) comparing surgical treatment against non-surgical, clinical management of flail chest injury. To minimise retrieval and publication bias, no limitations were set with regard to language, publication date or publication status. Studies were excluded if no results were available. Studies that included any non-flail or non-traumatic rib fractures were excluded. Any studies including individuals aged < 18 years were also excluded. Letters, conference papers, reviews, abstracts and case reports were excluded, along with any observational studies. The reference lists of relevant review articles were examined for any additional studies that may have been missed.

### Risk of Bias

The methodological quality of each of the studies, looking for any bias, was assessed using the Cochrane Risk of Bias (RoB) tool. Data were extracted and summarised using a data extraction form. The form included data on the characteristics of each study, information on participants, the interventions and the outcome measures.

### Statistical analysis

Between-study heterogeneity assessed by the *I*^2^ value, along with an estimate of the between-study variance in a random-effects meta-analysis, Tau^2^. The analysis has been carried out in *R* version 3.6.2 using the ‘meta’ package version 4.13. Graphically, data were presented as forest plots.

## Results

**Fig. 1:**
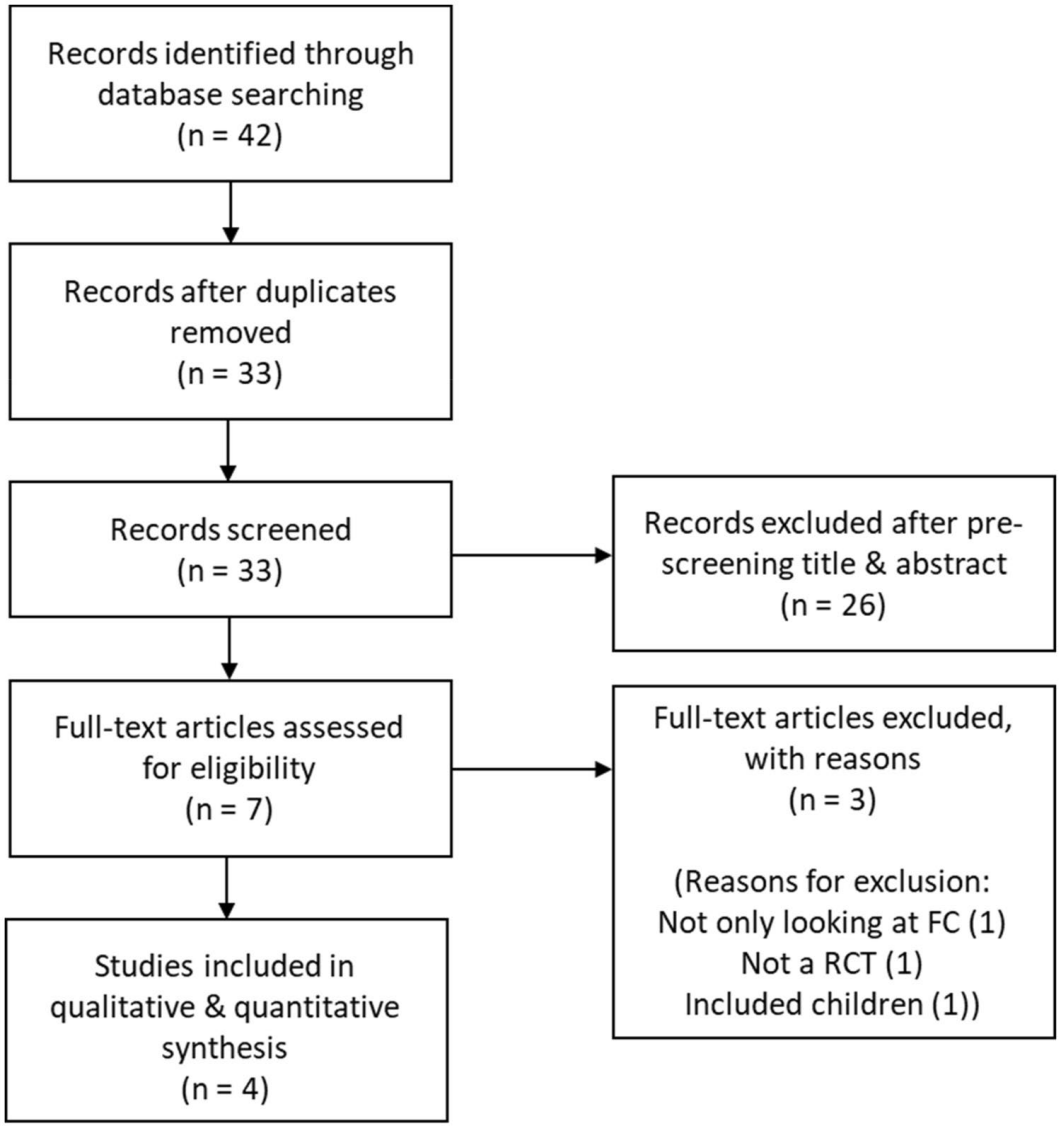
Prisma flow chart illustrating the literature search process

The search identified 42 studies in total that were retrieved from the electronic databases (Fig. 1). After removing duplicates, 33 studies were screened by title and abstract and 26 excluded. Full text articles of seven studies were screened, of which four studies met the inclusion criteria and were subsequently included in this systematic review.

### Studies and patients

The retrieval strategy is displayed in Fig. 1. The four studies (see Supplementary Table 2 for full characteristics of the studies) were prospective RCTs and involved 157 patients [11–14]. Liu et al. [13] reported a follow-up after 1 week and at 3 months post-discharge. Malhotra et al. [14] reported follow-up at 2 weeks, then at three and six months. Marasco et al. [12] also reported on 3- and 6-months follow-up reviews. Tanaka et al. [11] followed participants for 12 months. All patients were stabilised using mechanical ventilation prior to enrolment in the trials but other specifics of their injuries such as rib score, location of flail segment, sternum fracture, trauma score or other associated injuries were not reported.

**Fig. 2:**
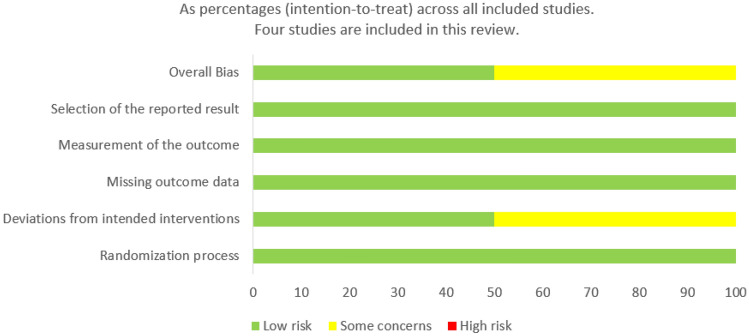
Risk of bias graph

**Fig. 3:**
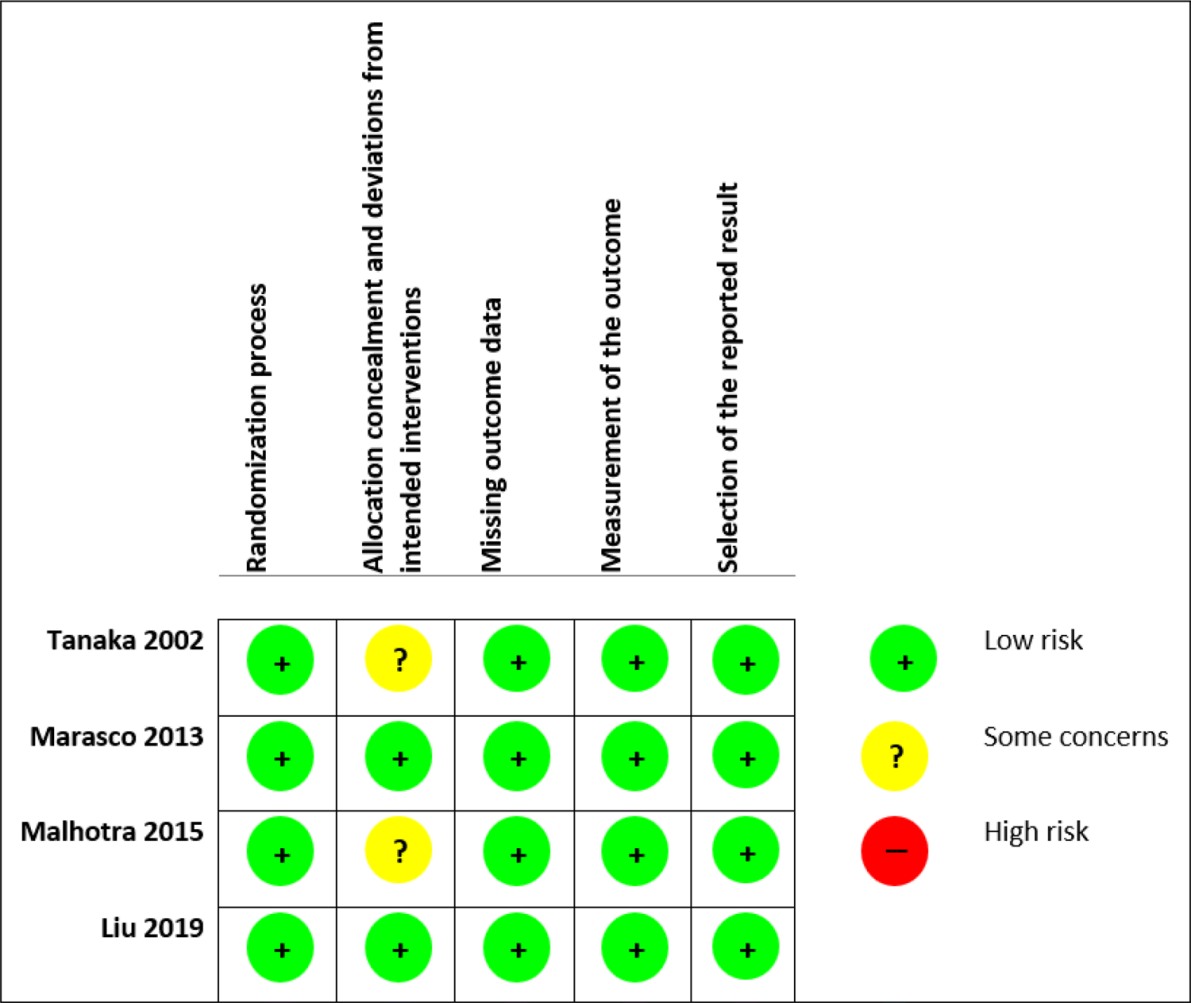
Risk of bias summary

Figures 2 and 3 show the summary from the risk of bias assessment. All studies reported on the generation of randomisation sequence, for this reason, they were all classified as having a low risk of bias. Methods of randomisation included a randomisation chart [11], computer-generated code using block randomisation with a block size of 4 [12], and random numbers balanced with a block size of 10 [13]. Two studies did not comment on whether an appropriate analysis was used to estimate the effect of assignment to intervention [11, 14]. As such, there was some concern regarding the potential impact (on the results) of the failure to analyse patients in the group to which they were randomised. In all other domains, all the studies were judged as having a low risk of bias.

### Types of interventions and outcomes measured

Tanaka et al. [11] included 37 patients in the trial and randomised them into surgical or non-surgical treatment groups. Patients were recruited from the hospital’s emergency room when they were admitted for chest trauma and FC; they also must have needed mechanical ventilation. Surgical fixation of the fractured ribs, using metal plates (Judet struts), were carried out on 18 patients and the remaining 19 were treated clinically, with non-surgical methods, in accordance with the standard of care of the institution. This happened to be orotracheal intubation and intermittent pressure ventilation. This study measured pneumonia, length of mechanical ventilation, tracheostomy, ICU stay, dyspnoea, costs, return to employment, long-term respiratory function, mortality, lung contusion, and at 6- and 12-months following injury, they used a questionnaire that recorded a number of other outcomes.

Marasco et al. [12] included 46 patients in the trial, with 23 of them randomised into the surgical group and the other 23 into the non-surgical group. All patients required invasive mechanical ventilation. For those assigned to the surgical management group, rib fractures between levels 3 and 10 were fixed using Inion resorbable (Inion OTPS) 6- or 8-hole plates and bicortical screws. Patients in the non-surgical group were treated with the standard of care for the institution, which in this case was mechanical ventilator management. This study evaluated the duration of mechanical ventilation, ICU stay and readmission, mortality, duration of stay in hospital, pneumonia, pneumothorax, chest deformity and tightness, failed extubation, tracheostomy, costs, return to employment and a quality-of-life questionnaire (36-item Short Form (SF-36)) that was given to participants at six months post-injury.

Malhotra et al. [14] included 24 patients in the trial; 13 were randomly assigned to the surgical group and 11 into the non-surgical group. Patients were recruited from the trauma unit with injuries that included either a stove-in-chest (adjacent rib fractures with at least two ribs pushed to a distance greater than the diameter of the rib that is pushed in) or a unilateral flail chest, which they defined as three or more ribs fractured at two places. All patients had to be on a ventilator. The patients in the surgical group were operated on within 72 h of ventilation (early fixation). Post-operatively, they received the same standard of care as the non-surgical group received post-injury, that is, the standard of care for the various institutions. The standard of care was not explicitly stated. The outcomes of interest in this study were total days on the mechanical ventilator, ICU length of stay, hospital length of stay and mortality. Forced vital capacity, forced expiratory volume one (FEV1) and results from a quality-of-life questionnaire (Rand 36 health survey) were evaluated at 3- and 6-months post-discharge. This study was also interested in how many participants were still using narcotics for pain control two weeks post-discharge.

Liu et al. [13] included 50 patients in the trial, with 25 of them randomised into the surgical group and the other 25 into the non-surgical management group. Poly-trauma patients with injury severity scores (ISS) of 16 or more were recruited from the trauma centre if FC was identified. U-plates were used for the surgical fixation of the fractured ribs and the routine management for the non-surgical group consisted of pain control, external fixation by chest splint or bandage, pulmonary physiotherapy, fibrobronchoscopic drainage and antibiotic administration. Intubation, thoracostomy and mechanical ventilation were also performed if needed; this is also true for the majority of randomised control trials in this review. This review evaluated mechanical ventilation days, length of stay in ICU and in hospital, in-hospital mortality, pneumonia, acute respiratory distress syndrome, sepsis, reintegration, tracheostomy, thoracic deformity, pain at admission and also after a week.

### Ongoing studies

Three ongoing studies were identified. The outcome of these studies should be included in a future update of this review if the eligibility criteria is met (NCT02635165; NCT02595593; NCT01367951).

### Primary outcome—length of stay in intensive care unit

A total of 157 patients were included in analysis of this outcome. All four studies report on the length of ICU stay, but unfortunately, because not all the studies reported results in the same format, we could not combine them for statistical analysis. Three of the studies show that significantly fewer days were spent in ICU by the participants who received surgery [11–13]. Length of ICU stay was reported in a few different ways in Marasco et al. [12], meaning that although the data were fairly interesting, it did not allow for combination with the other study data for meta-analysis. In contrast to the other three studies, Malhotra et al. [14] reported that the non-surgical group spent on average 10.1 fewer days in ICU (Table 1) [14].

**Table 1:** Intensive care unit length of stay (ILOS) as reported by the individual studies

Paper	Outcomes	Surgical group	Non-surgical group	*P* value
Tanaka et al. [11]		(*n* = 18)	(*n* = 19)	
		Days (mean ± SD)		
	Total length of TICU stay	16.5 ± 7.4	26.8 ± 13.2	< 0.05
	Length of stay in TICU after surgery	9.2 ± 5.2	N/A	
Marasco et al. [12]		(*n* = 23)	(*n* = 23)	
		Hours (mean ± SD)		
	ILOS pre-randomisation	61.6 ± 36.1	81.3 ± 84.2	0.31
	ILOS between pre-randomisation and surgery	49.4 ± 35.9	N/A	N/A
		Hours (median (IQR))		
	ILOS post-randomisation	285 (191–319)	359 (270–581)	0.03
	Total ILOS	324 (238–380)	448 (323–467)	0.03
Malhotra et al. [14]		(*n* = 13)	(*n* = 11)	
		Days (mean ± SD)		
	Total ILOS	23.1 ± 20.3	13.0 ± 6.1	
Liu et al. [13]		(*n* = 25)	(*n* = 25)	
		Days (median (IQR))		
	Overall ILOS	10 (7–12)	12 (9–15)	0.032
	ILOS with pulmonary contusion	11 (8–5)	11 (7–16)	0.28
	ILOS without pulmonary contusion	8 (6–11)	11 (7–14)	0.19

*ICU* intensive care unit, *TICU* trauma ICU, *SD* standard deviation, *IQR* interquartile range, *N/A* not applicable

### Length of stay in hospital

Tanaka et al. [11] did not report on the overall length of stay in hospital of their participants. The three other studies reported on the length of stay in hospital of their participants, but again, these were reported in different formats, so they could not be combined for meta-analysis. Two studies reported shorter lengths of stay in hospital for the surgical groups [12, 13]. Malhotra et al. [14] once again reported outcomes favouring the non-surgical group (Table 2).

**Table 2:** Hospital length of stay (HLOS) as reported by the individual studies

Paper	Outcomes	Surgical group	Non-surgical group	*P* value
Tanaka et al. [11]		(*n* = 18)	(*n* = 19)	
	HLOS	N/A	N/A	N/A
Marasco et al. [12]		(*n* = 23)	(*n* = 23)	
		(*d*, median, IQR)		
	HLOS	20 (18–28)	25 (18–38)	0.24
Malhotra et al. [14]		(*n* = 13)	(*n* = 11)	
		Days (mean ± SD)		
	HLOS	27.4 ± 18.7	20.8 ± 8.8	N/A
Liu et al. [13]		(*n* = 25)	(*n* = 25)	
		Days (median (IQR))		
	HLOS	21 (17–25)	22 (17–26)	0.44
	HLOS with pulmonary contusion	25 (20–28)	23 (19–27)	0.071
	HLOS without pulmonary contusion	17 (14–20)	18 (15–22)	0.056

*N/A* not applicable, *IQR* interquartile range, *SD* standard deviation

### Mortality

Of the 157 patients, there were seven in-hospital deaths reported. There was no statistically significant difference in the reported deaths between the surgical and the non-surgical groups. Two of the four studies reported deaths. From the seven people who died, one patient was mentioned to have died from sepsis, at day 92 post-injury following a massive blood transfusion [12]. The cause of death for the other six patients was not explicitly declared [13].

### Adverse effects of intervention

#### Infections

None of the studies reported on any kind of wound or bone infections post-injury.

#### Pain

Two papers reported on the differences in pain management between the two groups. Pain whilst coughing and deep breathing improved markedly in a week in the surgical group compared to the non-surgical, though there seemed to be no significant difference in pain whilst at rest [13]. Malhotra et al. [14] attempted to assess pain by looking at the number of patients still needing narcotics for pain control at the 2-week follow-up. A number of patients were lost to follow-up, one patient from the surgical group and three from the non-surgical group. Seven of the 12 in the surgical group, and 6 of 8 in the non-surgical group still required narcotics for pain management [14]. The other two studies did not report on any intervention-related pain [11, 12].

### Adverse progression in pulmonary status

#### Tracheostomy

**Fig. 4:**
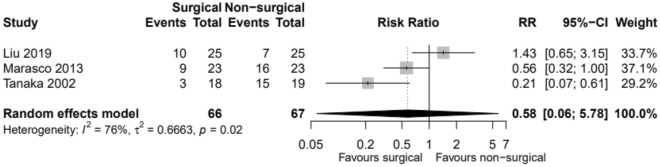
Incidence of the need for tracheostomy. *RR* relative risk, *CI* confidence interval

Three studies involving 133 patients reported on the use of tracheostomy [11–13]. In total, 60 events were reported (Table 3). Tracheostomy events were lower in the surgical group compared to the non-surgical group. There is little evidence to show there is a true difference in the risk of events between the two groups, and any observed effect may just be down to chance (Fig. 4).

**Table 3:** Need for tracheostomy post-injury

Paper	Outcomes	Surgical group	Non-surgical group	*P* value
Tanaka et al. [11]		(*n* = 18)	(*n* = 19)	
	Tracheostomy (day 7)	0	5	NS
	Tracheostomy (day 21)	3	15	< 0.05
Marasco et al. [12]		(*n* = 23)	(*n* = 23)	
	Tracheostomy	9	16	0.04
Malhotra et al. [14]		(*n* = 13)	(*n* = 11)	
	Tracheostomy	N/A	N/A	N/A
Liu et al. [13]		(*n* = 25)	(*n* = 25)	
	Tracheostomy	10	7	0.55

#### Pneumonia

Three studies, with a total of 133 patients reported on pneumonia (Table 4) [11–13]. All three of the studies clearly stated definitions for pneumonia. Tanaka et al. [11] defined pneumonia as purulent expectorate or end-tracheal aspirate from which known pathogens were grown (> 10^5^/mL), continued high fever (> 38 °C), leukocytes (> 10,000/µL) and recent infiltrate shadows on chest radiograph [11]. Marasco et al. [12] defined pneumonia as new infiltrate on X-ray, with positive sputum culture. Liu et al. [13] defined pneumonia as new infiltrate on chest x-ray, positive sputum culture and signs of systemic infection such as leucocytosis or fever.

**Table 4:** Incidence of pneumonia

Paper	Outcomes	Surgical group	Non-surgical group	*P* value
Tanaka et al. [11]		(*n* = 18)	(*n* = 19)	
	Pneumonia (day 7)	1	3	NS
	Pneumonia (day 21)	4	17	< 0.05
Marasco et al. [12]		(*n* = 23)	(*n* = 23)	
	Pneumonia	11	17	0.07
Malhotra et al. [14]		(*n* = 13)	(*n* = 11)	
	N/A	N/A	N/A	N/A
Liu et al. [13]		(*n* = 25)	(*n* = 25)	
	Pneumonia	12	20	0.038

**Fig. 5:**
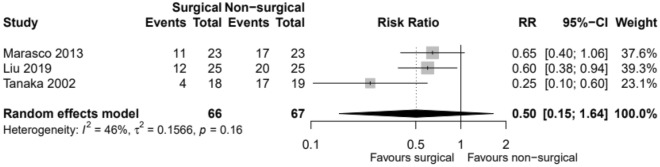
Incidence of pneumonia. *RR* relative risk, *CI* confidence interval

Out of 133 patients, 27 patients in the surgical group and 54 of the non-surgical patients acquired pneumonia. Events are lower in the surgical group compared to the non-surgical group (RR 0.50, CI 0.15; 1.64, *p* value 0.16). The risk of events is 50% lower in the surgical group compared to the non-surgical group. However, there is little evidence to show there is a true difference in the risk of events between the two groups, and any observed effect may just be down to chance (Fig. 5).

### Duration of mechanical ventilation

**Fig. 6:**
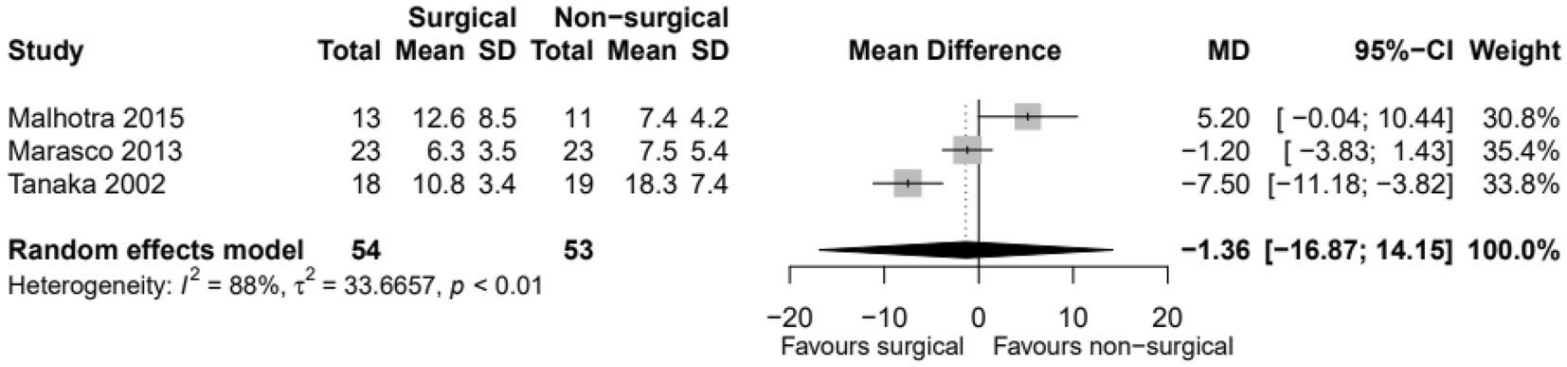
Duration of mechanical ventilation. *MD* mean difference, *CI* confidence interval

All four studies reported on the duration of mechanical ventilation (DOMV) (Table 5) [11–14]. Out of all 157 patients, the duration of mechanical ventilation was lower in the surgical group compared to the non-surgical group. There is little evidence to show there is a true difference in the duration of mechanical ventilation between the two groups, and any observed effect may just be down to chance (Fig. 6).

**Table 5:** Duration of mechanical ventilation (MV) as reported by the individual studies

Paper	Outcomes	Surgical group	Non-surgical group	*P* value
Tanaka et al. [11]		(*n* = 18)	(*n* = 19)	
		Days (mean ± SD)		
	Total MV	10.8 ± 3.4	18.3 ± 7.4	< 0.05
	MV after surgery	2.5 ± 3.2	N/A	
Marasco et al. [12]		(*n* = 23)	(*n* = 23)	
		Hours (mean ± SD)		
	Invasive MV post-randomisation	151.8 ± 83.1	181.0 ± 130.2	0.37
Malhotra et al. [14]		(*n* = 13)	(*n* = 11)	
		Days (mean ± SD)		
	MV	12.6 ± 8.5	7.0 ± 4.2	N/A
Liu et al. [13]		(*n* = 25)	(*n* = 25)	
		Days (median (IQR))		
	MV	7 (6–10)	9 (7–12)	0.012
	MV with pulmonary contusion	9 (7–13)	10 (7–16)	0.063
	MV without pulmonary contusion	5 (3–8)	8 (5–12)	0.015

*N/A* not applicable, *IQR* interquartile range, *SD* standard deviation

## Discussion

Currently, it is common for patients with FC resulting from severe thoracic trauma to be managed clinically, with non-surgical methods, but it is clear that the therapeutic effect has not been satisfactory [15]. There is increasing evidence that demonstrates the benefits of surgical intervention for such patients. We undertook a systematic review and meta-analysis of surgical fixation versus non-surgical management in the adult population, with a view to informing future practice. This review concludes that surgical intervention reduces the need for tracheostomy, reduces the time spent in the ICU following a traumatic flail chest injury and could reduce the risk of acquiring pneumonia after such an event.

Although surgical stabilisation is indicated by our systematic review of this small number of RCTs, when surgery for flail chest is indicated or the timing of surgery remains unclear. For example, in one study, patients were only enrolled if they were dependent on a ventilator with no prospect of successful weaning within the next 48 h [12]. Other studies did not mention the reasons why patients were included in their study or what criteria they used to judge when surgical fixation over non-surgical management was best. Likewise, the timing of surgical stabilisation in one study occurred 5 days after the initial injury [11]; however, other studies did not mention the timing of surgical intervention from initial injury. These two important parameters could have added variability to the outcomes of surgical management.

### Overall completeness and applicability of evidence and quality of the evidence

Although the number of patients included in the analyses was small (157 patients), surgery appeared to be beneficial for the main outcome, length of stay in ICU, and also for the secondary outcomes, pneumonia, tracheostomy and length of stay on mechanical ventilation. Considerable heterogeneity was detected for the duration of mechanical ventilation and tracheostomy outcomes to warrant caution when interpreting the results. It is possible that these results are down to chance. To be more confident in the conclusions of later analyses, larger multi-centre RCTs are needed. The quality of the included trials was generally good. They were all randomised appropriately, and allocation concealment was adequate for the type of intervention involved. Definition of outcomes such as pneumonia, by all the studies, adds to the strength of the quality of the evidence.

### Potential biases in the review process

Every effort was made to ensure that all studies that met the criteria were included in this review, but we cannot be certain as some RCTs may have never been published. It is always a possibility that some of the RCTs that have been included are the studies with stronger treatment effects. These things threaten the validity of this systematic review.

### Other studies

Lower mortality [16], lower risks of pneumonia [17–21], lower rates of chest deformity [21], a reduction in the need for tracheostomy and in the duration of mechanical ventilation and ICU stay [19, 21] have been seen as a result of surgical fixation in previous retrospective studies. The results of this systematic review are by and large consistent with the results seen in the previous retrospective studies and meta-analyses [22–24]. However, previous studies [23–25] only included three of the RCTs and reported on 61 patients whilst this study includes 4 RCTs with 157 patients in total. Hence, our study is more conclusive than these previous studies due to the larger number of patients involved in surgical fixation. Even so, more well-designed multi-centred RCTs are needed to produce strong evidence that could influence clinical practice.

### Author’s conclusions

Analyses of these fairly small RCTs, clearly show that there is some evidence that surgical fixation of the rib fractures leads to greater favourable outcomes than non-surgical management in FC. The majority of the evidence points to surgical treatment leading to a reduction in the rates of pneumonia and the need for tracheostomy. Although a significant difference in length of stay in hospital was not apparent in the evidence, there was shown to be improved resource utility by allowing patients to spend fewer days on mechanical ventilation and in ICU.

With regard to the implications for practice of the results of these studies, it is unlikely that these findings will lead to quick changes to recommendations in the standard of care in hospitals, especially here in the United Kingdom. Nevertheless, it should definitely start the conversation and highlight the urgent need for larger, multi-centred, high-quality RCTs.

## Supplementary Information

Below is the link to the electronic supplementary material.

Supplementary file1 (DOCX 27 KB)

## Data Availability

Not applicable.
