# Supplementary material for: Safety and effectiveness of surgical fixation versus non-surgical methods for the treatment of flail chest in adult populations: a systematic review and meta-analysis
Source: Eur J Trauma Emerg Surg. 2021 Feb 6;48(2):1025–34. doi: 10.1007/s00068-021-01606-2 (PMC7866961; doi:10.1007/s00068-021-01606-2)
Supplement: Supplementary file 1 — Supplementary file1 (DOCX 27 KB) [file 68_2021_1606_MOESM1_ESM.docx]

**Supplementary Table 1.** Database search process

| **Searching in Databases** | | | | |
| --- | --- | --- | --- | --- |
| **Name of the Database** | **Scopus** | **Search Query** | | **# of Results** |
| **Platform** | Elsevier | **1** | TITLE-ABS-KEY ( "Flail chest" ) AND ( LIMIT-TO ( LANGUAGE ,  "English" ) )  AND  ( LIMIT-TO ( PUBSTAGE ,  "final" ) )  AND  ( LIMIT-TO ( EXACTKEYWORD ,  "Flail Chest" ) ) | 1264 |
| **Year Coverage** | 1970 - 2020 | **2** | AND “surgery” OR “Surgical Procedure” | 610 |
| **Date of Search** | 22/07/2020 | **3** | AND “Conservative treatment” OR “conservative management” OR “non-surgical treatment” OR “non-surgical management” | 145 |
|  |  | **4** | AND “randomised control trial” OR “RCT” | 4 |
|  |  |  |  |  |
| **Name of the Database** | **Cochrane Central Register of Controlled Trials (CENTRAL, The Cochrane Library)** | **Search Query** | | **# of Results** |
| **Platform** | Cochrane group | **1** | Flail chest | 46 |
| **Year Coverage** | 1970 - 2020 | **2** | AND “surgery” OR “Surgical Procedure” | 18 |
| **Date of Search** | 22/07/2020 | **3** | AND “Conservative treatment” OR “conservative management” OR “non-surgical treatment” OR “non-surgical management” | 18 |
|  |  |  |  |  |
| **Name of the Database** | **PubMed** | **Search Query** | | **# of Results** |
| **Platform** | United States National Library of Medicine (NLM) | **1** | Flail chest | 11 |
| **Year Coverage** | 1970 - 2020 | **2** | AND “surgery” OR “Surgical Procedure” | 8 |
| **Date of Search** | 22/07/2020 | **3** | AND “Conservative treatment” OR “conservative management” OR “non-surgical treatment” OR “non-surgical management” | 1 |
|  |  | **4** | ~~AND “Conservative treatment” OR “conservative management” OR “non-surgical treatment” OR “non-surgical management”~~ | 8 |
|  |  |  |  |  |
| **Name of the Database** | **Embase OvidSP** | **Search Query** | | **# of Results** |
| **Platform** | Ovid | **1** | Flail Chest | 1177 |
| **Year Coverage** | 1974 - 2020 | **2** | limit 1 to (human and English language and randomized controlled trial) | 18 |
| **Date of Search** | 22/07/2020 | **3** | (Surgery or Surgical procedure).af. | 4799512 |
|  |  | **4** | limit 3 to (human and english language and randomized controlled trial) | 114417 |
|  |  | **5** | (Conservative treatment or conservative management or non-surgical treatment or non-surgical management).af. | 114800 |
|  |  | **6** | limit 5 to (human and english language and randomized controlled trial) | 3269 |
|  |  | **7** | 2 and 4 and 6 | 4 |
|  |  |  |  |  |
|  |  |  |  |  |
| **Name of the Database** | **Ovid MEDLINE(R) and In-Process & Other Non-Indexed Citations** | **Search Query** | | **# of Results** |
| **Platform** | Ovid | **1** | Flail chest.af. | 852 |
| **Year Coverage** | 1970 - 2020 | **2** | limit 1 to (english language and humans and randomized controlled trial) | 9 |
| **Date of Search** | 22/07/2020 | **3** | (Surgery or Surgical Procedure).af. | 3587011 |
|  |  | **4** | limit 3 to (english language and humans and randomized controlled trial) | 85525 |
|  |  | **5** | (Conservative treatment or Conservative management or Non-surgical treatment or Non-surgical management).af. | 47972 |
|  |  | **6** | limit 5 to (english language and humans and randomized controlled trial) | 1364 |
|  |  | **7** | 2 and 4 and 6 | 1 |
|  |  |  |  |  |
|  |  |  |  |  |
| **Name of the Database** | [**Clinicaltrials.gov (www.clinicaltrials.gov)**](http://www.clinicaltrials.gov/) | **Search Query** | | **# of Results** |
| **Platform** |  | **1** | Surgery \| Interventional Studies \| Flail Chest \| Adult, Older Adult | 7 |
| **Year Coverage** |  |  |  |  |
| **Date of Search** | 22/07/2020 |  |  |  |

**Supplementary Table 2.** Characteristics of the studies

| Tanaka et al., 2002 [11] | | |
| --- | --- | --- |
| Methods | Study design | randomised control study |
|  | Number of centres | Single |
|  | Setting | Kyorin University hospital, Tokyo, japan |
|  | Study period | April 1992 - March 1998 |
|  | Sample size | 37 |
|  | Follow up | 12 months |
| Participants | Allocation (Surgical/Non-operative) | 18 |
|  |  | 19 |
|  | Sex (M/F) | 26 / 11 |
|  | Age (mean +/- SD) (Surgical / Non-operative) | 43 +/- 12 |
|  |  | 46 +/- 9 |
|  | Inclusion criteria | Chest trauma, flail chest |
|  | Exclusion criteria |  |
| Interventions | Surgical | Surgical stabilisation |
|  | Non-operative | Internal pneumatic stabilisation |
| Outcomes |  | Incidence of pneumonia, incidence of tracheostomy, length of mechanical ventilation, length of stay in intensive care unit, total medical expense, long term respiratory function by spirometer, subjective dyspnoea, and a questionnaire |
|  |  |  |
| Marasco et al., 2013 [12] | | |
| Methods | Study design | randomised control study |
|  | Number of centres | Single |
|  | Setting | The Alfred Hospital - Adult Major Trauma Centre, Victoria, Australia |
|  | Study period | January 2007 - December 2011 |
|  | Sample size | 46 |
|  | Follow up | 6 months |
| Participants | Allocation (Surgical/Non-operative) | 23 |
|  |  | 23 |
|  | Sex (M/F) | 40 / 6 |
|  | Age (mean +/- SD) (Surgical / Non-operative) | 57.8 +/- 17.1 |
|  |  | 59.3 +/- 10.4 |
|  | Inclusion criteria | presence of a flail segment defined as 2 3 consecutive ribs fractured in more than one place, producing a free-floating segment Of chest wall, diagnosis was made clinically With confirmation on 3-dimensional computed tomography imaging, ventilator dependent With no prospect Of successful weaning within the next 48 hours |
|  | Exclusion criteria | aged > 80 years, spinal injuries, open rib fractures with soiling or infection, sepsis, severe traumatic brain injury (Glasgow Coma Scale < 10 at the scene of accident or at presentation to the hospital, as its management may have received priority over the study protocol), and uncorrected coagulopathy |
| Interventions | Surgical | only rib fractures between the levels Of ribs 3 and 10 were fixed. Ribs fractured more than once were usually addressed by fixing 1 fracture per rib, converting a flail segment to simple fractured ribs.  Ribs with a single fracture were not fixed unless there was gross deformity mandating intervention. Anterior and lateral rib fractures were preferentially fixed over posterior rib fractures |
|  | Non-operative | Mechanical ventilation |
| Outcomes |  | Duration of mechanical ventilation; length of ICU stay; pneumonia, pneumothorax, intercostal catheterisation, tracheostomy, ICU readmission, duration of hospital stay, cost assessment of operation. |
|  |  |  |
| Malhotra et al., 2015 [14] | | |
| Methods | Study design | randomised control study |
|  | Number of centres | N/A |
|  | Setting |  |
|  | Study period | September 2010 - August 2014 |
|  | Sample size | 24 |
|  | Follow up | 6 months |
| Participants | Allocation (Surgical/Non-operative) | 13 |
|  |  | 11 |
|  | Sex (M/F) | 20/4 |
|  | Age (Surgical / Non-operative) | N/A |
|  |  | N/A |
|  | Inclusion criteria | Adults >21 years old and <75 years old. Unilateral flail chest (>3 ribs fractured at two places) or Contiguous rib fractures with at least 2 ribs pushed in > the rib diameter of the pushed in rib |
|  | Exclusion criteria | Patient unlikely to survive due to the trauma or age or multiple co-morbidities; Stove-in chest patients that do not require early (less than or equal to 48 hours of injury) ventilatory support; Bilateral flail chest; Sternal flail; P/F ratio < 200:1 over a period of greater than or equal to 6 hours while on the ventilator; Other injuries that will likely prolong tracheal intubation and mechanical ventilation e.g. significant head injury resulting in low GCS (Glasgow Coma Score, a scale used to assess the central nervous system in patients who have undergone trauma), spinal cord injury resulting in paralysis of some or all of the respiratory muscles etc. These are merely examples. It is in the opinion of the investigator/surgeon what injuries would prolong tracheal intubation; Any contra-indication to surgery including severe immunosuppression or severe chronic disease making elective surgery dangerous in the opinion of the surgeon; Inability to proceed with any aspect of critical care due to personal beliefs, living will etc e.g. non acceptance of blood products; Inability to obtain informed consent; Subject's refusal for follow up; Pregnant women; Prisoners; Any other reason for which the potential subject is not a good candidate, in the opinion of the investigator. |
| Interventions | Surgical | Operative rib fixation |
|  | Non-operative | a. Ventilatory support b. Timing of extubation (removal from ventilator): c. Analgesia: institution should provide adequate analgesia utilizing available resources including oral, parenteral, epidural, local nerve blocks etc., d. Chest physical therapy, e. Postural drainage, f. Incentive spirometry - after extubation. |
| Outcomes |  | total days on ventilator, ICU length of stay, hospital length of stay; Mortality; quality of life; pulmonary function (FVC, FEV1); still on narcotics at post-discharge follow up. |
|  |  |  |
| Liu et al., 2019 [13] | | |
| Methods | Study design | randomised control study |
|  | Number of centres | Single |
|  | Setting | Tongji Hospital, Trauma Centre, Tongji Medical College of Huazhong University of Science and Technology, Wuhan, China |
|  | Study period | January 2015 - July 2017 |
|  | Sample size | 50 |
|  | Follow up | 3 months |
| Participants | Allocation (Surgical/Non-operative) | 25 |
|  |  | 25 |
|  | Sex (M/F) | 41/6 |
|  | Age (mean (range)) (Surgical / Non-operative) | 42 (25 - 58) |
|  |  | 39 (24 - 56) |
|  | Inclusion criteria | polytrauma with injury severity score (ISS) of 16 or more, flail chest |
|  | Exclusion criteria | age, death within 48 h, cervical spinal cord injury with paralysis, severe head injury (abbreviated injury Score [AIS] of head >3 and Glasgow Coma Scale [GCS] < 8), uncorrected coagulopathy,  and pre-existing cardiac or pulmonary conditions |
| Interventions | Surgical | Rib open reduction and internal fixation (ORIF) |
|  | Non-operative | pain control, external fixation by chest splint or bandages, pulmonary physiotherapy, fibrobronchoscopic drainage, antibiotics, (intubation, thoracostomy and mechanical ventilation if needed). |
| Outcomes |  | in-hospital mortality, incidence of complications (pneumonia. Acute respiratory distress sydrome and sepsis), and resources utility (MV days [MVDS], ICU length Of Stay [ILOS], and hospital length Of Stay [HLOS]) |
